# Collection of triatomines from sylvatic habitats by a *Trypanosoma cruzi*-infected scent detection dog in Texas, USA

**DOI:** 10.1371/journal.pntd.0010813

**Published:** 2023-03-20

**Authors:** Devin M. Christopher, Rachel Curtis-Robles, Gabriel L. Hamer, Justin Bejcek, Ashley B. Saunders, Walter D. Roachell, Thomas Leo Cropper, Sarah A. Hamer

**Affiliations:** 1 Independent Contractor, Columbus, Ohio, United States of America; 2 Department of Veterinary Integrative Biosciences, School of Veterinary Medicine & Biomedical Sciences, Texas A&M University, College Station, Texas, United States of America; 3 Department of Entomology, Texas A&M University, College Station, Texas, United States of America; 4 Department of Small Animal Clinical Sciences, School of Veterinary Medicine & Biomedical Sciences, Texas A&M University, College Station, Texas, United States of America; 5 Public Health Command Central, JBSA-Fort Sam Houston, San Antonio, Texas, United States of America; 6 Wilford Hall Ambulatory Surgical Center, Joint Base San Antonio, San Antonio Texas; Fundacao Oswaldo Cruz Instituto Rene Rachou, BRAZIL

## Abstract

**Background:**

Triatomine insects, vectors of the etiologic agent of Chagas disease (*Trypanosoma cruzi*), are challenging to locate in sylvatic habitats. Collection techniques used in the United States often rely on methods to intercept seasonally dispersing adults or on community scientists’ encounters. Neither method is suited for detecting nest habitats likely to harbor triatomines, which is important for vector surveillance and control. Furthermore, manual inspection of suspected harborages is difficult and unlikely to reveal novel locations and host associations. Similar to a team that used a trained dog to detect sylvatic triatomines in Paraguay, we worked with a trained scent detection dog to detect triatomines in sylvatic locations across Texas.

**Principle methodology/Findings:**

Ziza, a 3-year-old German Shorthaired Pointer previously naturally infected with *T*. *cruzi*, was trained to detect triatomines. Over the course of 6 weeks in the fall of 2017, the dog and her handler searched at 17 sites across Texas. The dog detected 60 triatomines at 6 sites; an additional 50 triatomines were contemporaneously collected at 1 of these sites and 2 additional sites without the assistance of the dog. Approximately 0.98 triatomines per hour were found when only humans were conducting searches; when working with the dog, approximately 1.71 triatomines per hour were found. In total, 3 adults and 107 nymphs of four species (*Triatoma gerstaeckeri*, *Triatoma protracta*, *Triatoma sanguisuga*, and *Triatoma indictiva*) were collected. PCR testing of a subset revealed *T*. *cruzi* infection, including DTUs TcI and TcIV, in 27% of nymphs (n = 103) and 66% of adults (n = 3). Bloodmeal analysis of a subset of triatomines (n = 5) revealed feeding on Virginia opossum (*Didelphis virginiana*), Southern plains woodrat (*Neotoma micropus*), and eastern cottontail (*Sylvilagus floridanus*).

**Conclusion/Significance:**

A trained scent detection dog enhanced triatomine detections in sylvatic habitats. This approach is effective at detecting nidicolous triatomines. Control of sylvatic sources of triatomines is challenging, but this new knowledge of specific sylvatic habitats and key hosts may reveal opportunities for novel vector control methods to block the transmission of *T*. *cruzi* to humans and domestic animals.

## Author summary

Triatomine insects, also known as ‘kissing bugs,’ are vectors of *Trypanosoma cruzi*, the parasite that causes Chagas disease in humans, dogs, and other mammals. Triatomines are found throughout the Americas, but for many species, little is known about where these blood-sucking insects spend their early life stages. Knowing more is important to vector control initiatives aimed at preventing Chagas disease. Scent detection dogs have been trained to detect many pests, including one study in Paraguay of a dog trained to detect triatomines. In this study, we used a dog to detect triatomines in their natural environments throughout Texas. Over 6 weeks, the dog identified 60 triatomines at 6 different sites; an additional 50 triatomines were collected without the dog’s assistance. More triatomines were collected per hour when the dog was searching when compared to only humans searching. Of all the triatomine nymphs collected, 27% were positive for *T*. *cruzi*, and bloodmeal analyses revealed kissing bugs had fed on Southern plains woodrat, opossum, and eastern cottontail. This study outlines a strategy that can be replicated in the United States to enhance the detection and control of habitats where triatomines spend their early life stages.

## Introduction

Chagas disease (American trypanosomiasis) is caused by the vector-borne protozoan parasite *Trypanosoma cruzi*. Approximately 5.7 million people across Latin America [1] and 300,000 people in the United States (US) [2] are estimated to be infected with *T*. *cruzi*. Infection also occurs in a variety of mammalian species in the US [3], including dogs, which are burdened with disease throughout the southern US [4–10]. The triatomine insect vectors of *T*. *cruzi* are found throughout the Americas, including the southern half of the US, where 11 species have been documented [11]. Limited Chagas disease treatments leave vector control as a primary means of reducing the burden of disease. Although many countries have executed successful intra-country and inter-country vector control programs throughout the past several decades [12,13], elucidating the sylvatic sources of triatomine populations remains a critical component to understanding triatomine natural history and further improving vector control efforts.

In contrast to some triatomine species found in South and Central America that readily colonize houses and transmit *T*. *cruzi* to people in their homes (e.g., *Triatoma infestans* and *Rhodnius prolixus*), it is rare for the triatomine species found in the southern US to colonize a house [14,15]. Triatomines in the US exist mainly in the sylvatic environment, where they transmit *T*. *cruzi* among wildlife reservoirs [3]. They occasionally disperse to domestic and peridomestic environments, where they pose a risk of transmission to humans and domestic animals. For example, triatomines have recently been collected by researchers and community scientists from houses, dog kennels, porches, and garages [16–19]. However, these collections were mainly of adult triatomines and predominantly during the summer months when the adult insects were dispersing by flight from their nidal habitats. These findings reveal little about the sylvatic habitats that support nymphal development. Although triatomines have been frequently collected from wood rat (*Neotoma* spp.) middens across the southwestern US [20–27], relatively little is known about other bloodmeal sources triatomines in the US may successfully and/or commonly utilize, particularly during their flightless nymphal stages. Manual searching for triatomines in sylvatic habitats is time- and labor-intensive, and success may be affected by the searcher’s familiarity with likely locations of triatomine habitat. For example, conspicuous harborage sites [8] and *Neotoma* woodrat middens [28] may be more likely to be searched, although less conspicuous triatomine nidal sites likely exist.

Scent detection dogs possess the ability to discern an incredible variety of odors and have been trained to identify diverse targets, including hidden explosives, fire accelerants, hazardous chemicals, illegal drugs, humans (search-and-rescue), drowning victims, invasive species, and endangered species [29]. Medical scent detection dogs are increasingly used for pre-screening of humans for infectious agents (e.g., SARS-CoV-2 [30,31]; malaria [32]; *Clostridium difficile* [33,34]) or chronic medical conditions (e.g., diabetes alert dogs [35]). Detection dogs have also been trained and used for the detection of insects such as bed bugs (*Cimex lectularius*) [36,37] and invasive insects including eastern subterranean termites (*Reticulitermes flavipes*) [38], spongy moths (*Lymantria dispar*) [39], brown marmorated stink bugs (*Halyomorpha halys*) [40], and spotted lantern fly (*Lycorma delicatula*) egg masses [41]. In addition, dogs have been trained to detect primary screwworm (*Cochliomyia hominivorax*) larvae and animals infected with screwworms [42], as well as animals infected with sarcoptic mange [43], demonstrating application of detection dogs for disease detection and control. Of most relevance here, a study in Paraguay used a detection dog to identify sylvatic sources of *Triatoma infestans* in the Paraguayan Chaco [44]. In this region, the sylvatic existence of *T*. *infestans*, the main vector of Chagas disease throughout South America’s Southern Cone, poses major problems to domestic control if sylvatic insects serve as a source population [13,45–47]. At sites where previous triatomine collection efforts using light traps and Noireau traps were largely unsuccessful, the use of the trained dog resulted in collecting 70 triatomines over a 4-month period, including samples with a novel *T*. *infestans* mitochondrial cytochrome B gene haplotype, from a variety of sylvatic sources [44].

We aimed to apply the concept introduced by the Paraguay group for dog detection of triatomines in different settings across Texas. Our objectives were to (i) demonstrate that a scent detection dog could be used to detect triatomines in sylvatic habitats in the US; (ii) collect live insects to supplement a laboratory colony of triatomines; and (iii) determine the infection prevalence, *T*. *cruzi* genetic strain(s), and bloodmeal hosts of collected triatomines.

## Materials and methods

### Ethics statement

All dog handling was completed in adherence with animal use protocols approved by Texas A&M University Institutional Animal Care and Use Committee under protocol number 2015–0289.

### Site characterization

**Fig 1:**
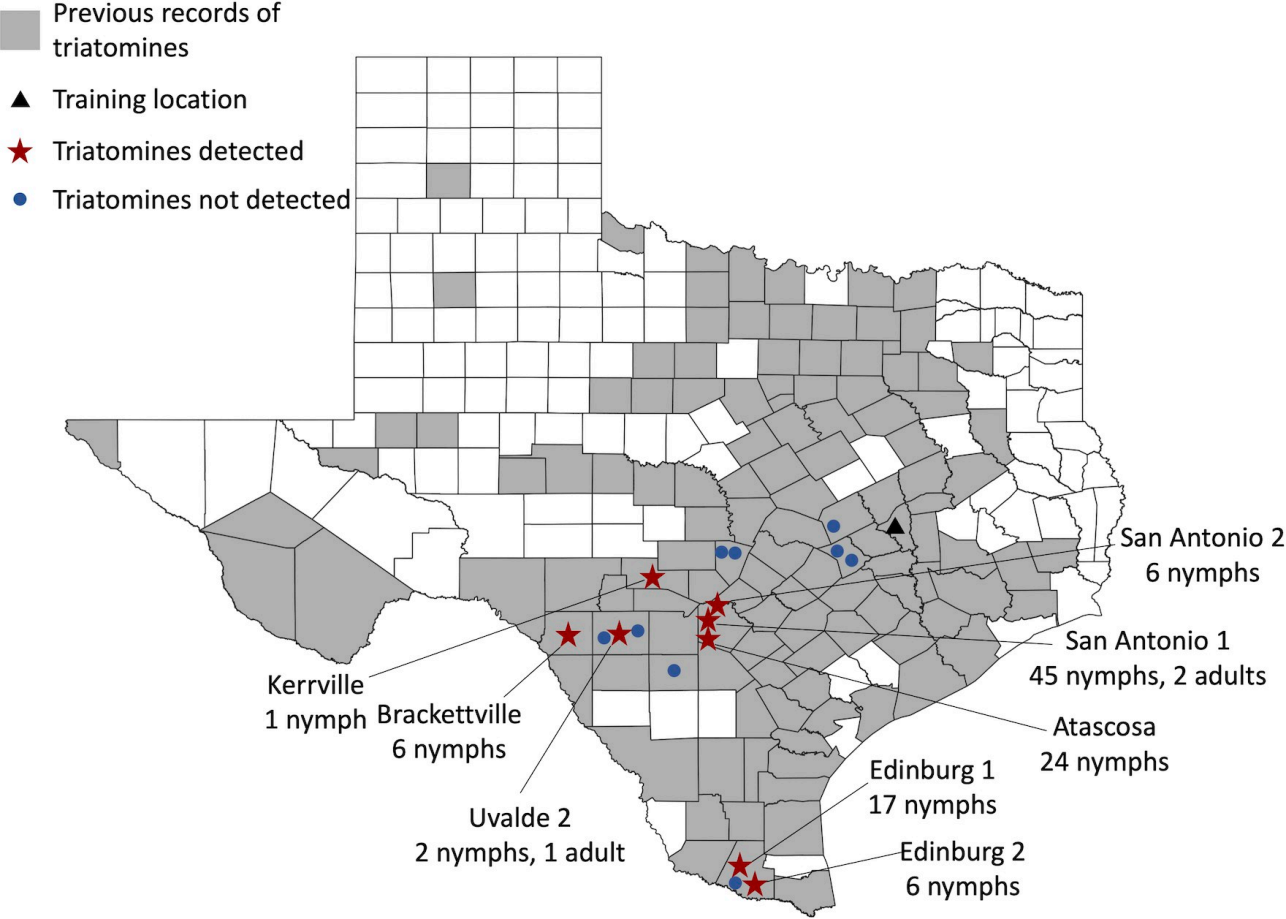
Training and field sites across Texas where triatomine scent detection efforts by a trained dog were conducted. Dog scent detection efforts were conducted at locations where kissing bugs had been submitted by community scientists or collected by our field team previously. Initial training for this study took place in College Station (triangle). Triatomines were found at 8 of the 17 sites searched. Previous records of triatomines are based on our field collections and submission to the Kissing Bug Community Science Program [18]. Base map created using QGIS [49] with Texas counties data file from https://data.texas.gov/dataset/Texas-Counties-Map/48ag-x9aa [50].

Field work was conducted in Texas, US, where seven species of triatomines have been documented [11]. Initial triatomine scent detection training was completed in College Station, Texas. Attempts to detect triatomines took place at 17 additional sites throughout Texas (Fig 1); sites were selected from areas with known triatomine presence, based on submissions to our community science program [48] and our own field work [18]. This study was conducted in the late fall (October 17th–December 1st, 2017), outside of the peak adult triatomine flight and collection period in Texas (generally mid-April through mid-October [18,27]), when non-traditional methods of triatomine collection from sylvatic habitats are necessary for successful collection.

### Dog health history and monitoring

As a dog trained to detect triatomines may physically contact *T*. *cruzi*-infected triatomines and their feces, there may be a high risk of parasite transmission to the dog. As such, we conducted our study with a previously (naturally) infected, but asymptomatic, dog.

‘Ziza,’ a German Shorthaired Pointer born in July 2014, was originally trained as part of the Transportation Security Administration (TSA) program by the US government. Ziza had trained as an Explosive Detective Canine to screen baggage, terminals, and mass transit vehicles. The training for such dogs typically spans a period of approximately 100 days, starting with training with just the dog and then continuing with their assigned handler. The training program uses a reward-based system in which dogs are rewarded when near the source of a target odor. Training starts unblinded (handler is aware of the source) and progresses to single blind (an evaluator is aware of the source, but handler is not) and then double blind (neither handler nor evaluator is aware of source) so as to reduce the possibility of the dog responding to the influence of people in the room. These dogs are estimated to value in excess of $50,000 USD once trained, given the costs of procurement (often overseas), quarantine, medical care, training, housing, feeding, and attrition (Brian D. Farr, MAJ, VC, DVM, MSTR, Diplomate ACVPM, personal communication).

Chagas disease is a known threat to the health of government-owned working dogs trained in Texas, where triatomine encounters are likely common; studies have revealed seroprevalence of 8% or more in dogs working in Texas and New Mexico [7,8]. As part of a routine semi-annual exam in June 2016, medical records showed Ziza tested positive via PCR for *T*. *cruzi* and positive for anti-*T*. *cruzi* antibodies on an indirect fluorescent antibody (IFA) test (titer value of 8192); IFA testing over the next six months remained positive. Thoracic radiographs and ECGs during these six months did not reveal any clinically significant abnormalities. Upon diagnosis with asymptomatic *T*. *cruzi* infection, the dog was retired from her work and was adopted several months later privately (by author DMC) in January 2017, for the purpose of training for triatomine detection. The owner noted no deviations from normal physical activity.

During this study, several tests were performed to monitor potential impacts of the existing *T*. *cruzi* infection on Ziza’s health. Pre- and post-field work (October and December, 2017), blood was collected from the dog for testing. *T*. *cruzi* antibody testing was performed on serum (pre-study) or plasma (post-study) using IFA testing at the Texas Veterinary Medical Diagnostic Laboratory (College Station, TX). DNA extracted from blood buffy coat layer was subjected to real-time PCRs for *T*. *cruzi* DNA detection and DTU typing [51–54] using variable DNA concentrations (neat, 2X, 1:10 dilution) to optimize chances of assigning a DTU. A 5-lead continuous read ambulatory ECG ‘Holter’ monitor (LabCorp, Burlington, NC) was applied for 48 hours during the final week of field work. Tracings obtained from the ECG were recorded at 25mm/s using a lead configuration of V1, V2 and V5, then transferred to LabCorp for automatic analysis. Tracings were reviewed by a board-certified veterinary cardiologist (author ABS).

### Training of scent detection dog

Eight weeks before beginning her work on triatomines, Ziza entered a pre-training period to review prior training and learn ‘digging’ as a secondary indication to indicate exact location upon finding. A ‘sit’ remained the dog’s official primary indication of a find. The dog was worked on a 6-meter lead.

Practice with triatomines and their scents began in October 2017. A total of 36 unique training trials were completed over the course of six days; each trial included between 1 and 25 repeats to allow for reinforcement. Trainings lasted between 5 and 25 minutes each. Laboratory-raised, uninfected *T*. *gerstaeckeri* nymphs and filter paper heavily contaminated with uninfected feces from the triatomine colonies at Texas A&M University were the primary training samples, but both live and dead *T*. *gerstaeckeri* adults were also used. Trials used a total of 7 different combinations of: fecal paper, 1 adult triatomine, and up to 3 triatomine nymphs. For biocontainment, live triatomines were placed inside air-permeable, but escape-proof, containers before use in training exercises. Containers were of nine varied sizes and materials, such as glass, plastic, and metal, so that the dog would not associate a particular container with the triatomine scent.

The first day of training consisted primarily of imprinting activities and simple practice searches in which a contained live triatomine was presented, and the dog was rewarded with a tennis ball for sniffing the triatomine. The complete search behavior chain was taught by progressive shaping and positive reinforcement [55,56]. Having previous scent detection experience, the dog quickly learned the new odor and could perform simple indoor searches before the end of the first day. The level of difficulty of the search problems was increased incrementally, until the dog could search consistently for longer than 10 minutes and reliably detect the test sample(s) in a search area at least 300m^2^. The first day and a half, training searches were performed indoors in a closed uncluttered garage with few hiding places. During the second and third days, training searches took place in an outdoor yard approximately 1,500m^2^ with gravel, leaves, and short grass. The fourth and fifth days, training searches took place in tall grass and wooded areas approximately 200,000m^2^, with the dog searching 2,000–4,000m^2^ at a time. By the end of the sixth day (a total of approximately 19 hours of the dog actively training), Ziza could reliably and quickly locate live triatomines in containers placed inside hollow logs, underneath small piles of brush or lumber, and lightly buried under dirt and leaves (to closely resemble triatomine natural habitat localities).

While in the field, reinforcement training was done 19 of the 46 days of the study, at least every 8 days. Trainings were 3–45 minutes with live and/or dead nymphs (up to 8) and/or adults (up to 6), as well as wood or paper that had been in a container with nymphs and was contaminated with their feces. Container types included small plastic containers, pill bottles, glass beakers, a glass salt shaker, a wooden box, tubes, gauze, or simply the triatomines (dead specimens collected locally).

### Field work

**Fig 2:**
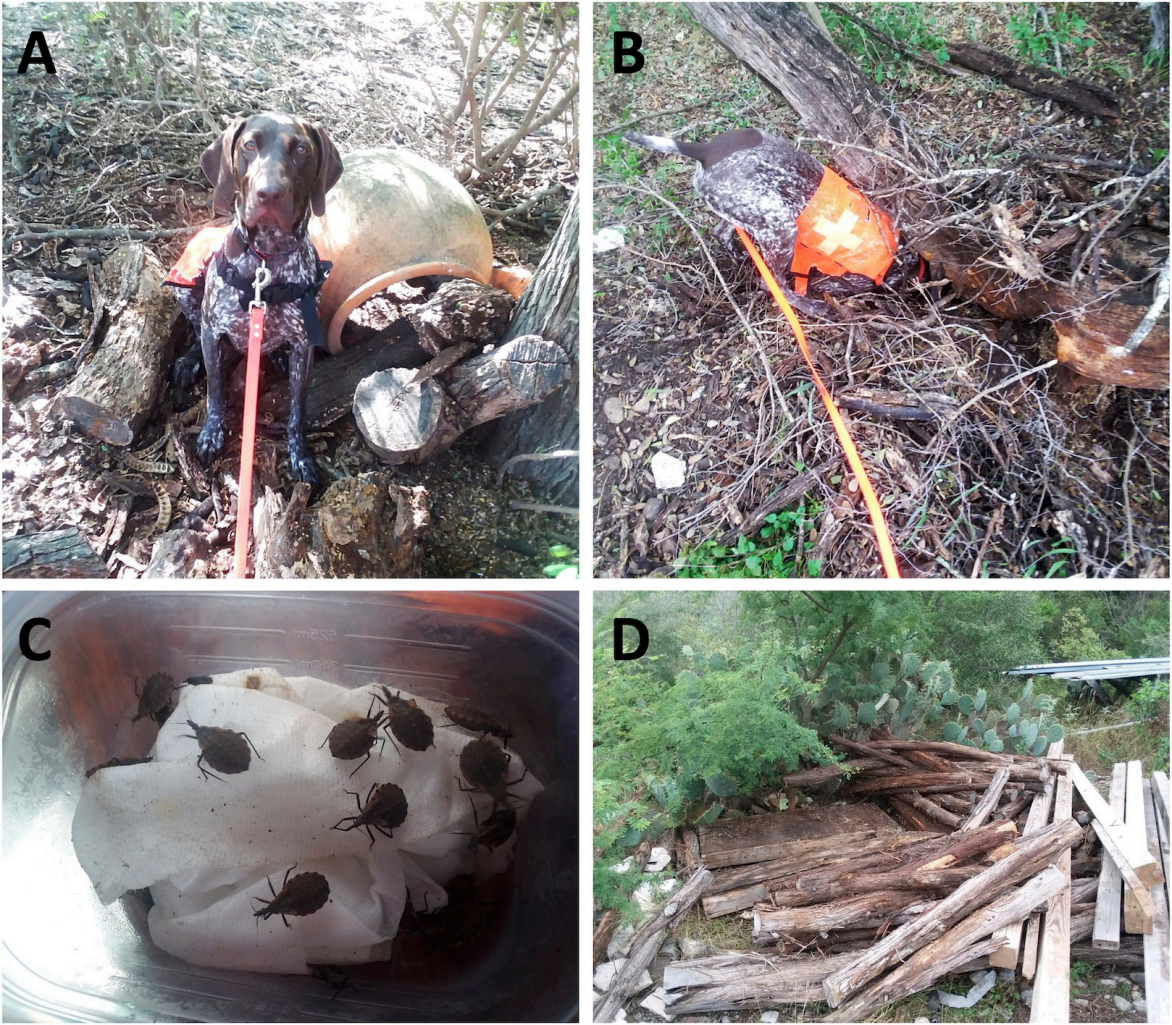
Triatomine detection dog in the field. (A) ‘Sit’ was the primary indication of a detection. (B) ‘Dig’ was the secondary indication of a detection. (C) Blood fed nymphs found at a south Texas site, in a small pile of railroad ties in the corner of a horse pen where rabbits had been seen. (D) Woodpile where nymphs were found.

After one week of training, Ziza, her handler, and at some sites, an assistant, visited 17 field sites across Texas (Fig 1). Sites were visited in October—December (2017) for two reasons: 1) weather is cooler and less stressful for the dog, and 2) dispersal of adult triatomines is minimal, so the dog would hopefully be detecting sylvatic sources containing nidicolous nymphs. Most days began with a practice problem using training samples as outlined above. The search area was then checked over by the handler and assistant prior to working the dog to identify hazards and choose the optimal search locations. Candidate search areas with obvious hazards such as broken glass, sharp metal, or dense patches of spiny plants were searched only by the human team members. The dog searched habitats including, but not limited to, woodpiles (including some piles of telephone poles), heavy (non-spiny) vegetation, downed logs, and likely animal nests (holes or depressions with woody debris, leaves, paper, cardboard, and other ‘nesting materials’ present, as well as animal feces–likely rodent–in the immediate vicinity). Ziza was encouraged to check each of the search areas as the handler and/or assistant turned items over and created room for her to access areas of interest. When the dog displayed her primary (sit) and/or secondary (dig) indication (Fig 2A and 2B), the location was marked for further inspection by the handler/assistant. Searching sessions continued so long as the dog appeared to be on task and did not display signs of discomfort such as heavy panting, slow movement, or lying down; searching ceased when all suitable search areas were exhausted. When conditions allowed (e.g., temperature lower than 21° C, search area fully shaded, and searching primarily woody debris and logs) the dog could work as long as two hours and rest as little as 20 to 30 minutes before working again. When conditions were difficult (e.g., temperature higher than 30° C, search area in full sun, or searching primarily areas with thorny plants or other dangers) the dog could only search for 15 to 20 minutes and then was given a minimum of 1 hour to rest before working again.

Triatomine findings were classified as one of three types: 1) triatomines located solely by dog, which did not require the handler or assistant to move anything in order for the dog to indicate the presence of triatomines, although the handler/assistant may have had to move the item to see and collect the triatomines; 2) triatomines located by dog with a human assist, which required the handler and/or assistant to move a large object or turn over one or more objects before the dog indicated the presence of triatomines; 3) triatomines located by human team members with no assistance by dog. Searches by humans, without the dog, were done primarily during daylight hours when it was very hot and while the dog rested, this included areas where the dog may have showed interest but had not indicated (sit and/or dig). Human searching included flipping over logs, railroad ties, and lumber/debris from piles; no intensive digging or movement of very large items was done.

### Triatomine infection with *T*. *cruzi* and bloodmeal analysis

Adult triatomines were identified to sex and species using morphologic features [14]. Most nymphs entered the laboratory colony for use in future projects and were monitored to determine the species once they molted into adults. A subset of twelve nymphs were subjected to PCR of the cytochrome b genetic region to determine species [57]. DNA extracted from hindgut sample (KingFisher Cell and Tissue DNA kit, Thermo Fisher Scientific, Waltham, MA) was subjected to PCR amplification and Sanger sequencing of the mitochondrial cytochrome b gene using previously published protocols [18]. Chromatograms were visually inspected for quality in MEGA version 11 [58] and sequences were compared to existing sequences in GenBank (https://www.ncbi.nlm.nih.gov/genbank/) [59] using BLAST [60] (https://blast.ncbi.nlm.nih.gov/Blast.cgi).

A subset of 106 specimens was subjected to *T*. *cruzi* testing. Two adult and ninety-nine live nymphal specimens that entered our laboratory colony were housed individually until they generated a fecal spot on filter paper that could be tested for *T*. *cruzi* DNA. One adult and four nymphal specimens that did not enter the laboratory colony were prepared and dissected as previously described [18], including determination of bloodmeal scores (scores of 1–5 indicating no blood to large amount of blood, respectively) and bloodmeal analysis. DNA was extracted from fecal spot or hindgut samples (KingFisher Cell and Tissue DNA kit). Each set of DNA extractions included no-template controls. Presence of *T*. *cruzi* DNA was detected via a quantitative PCR using Cruzi 1/2/3 [51–53]; confirmation of infection and determination of *T*. *cruzi* DTU was completed using an SL-IR target probe-based quantitative PCR method [51,54]. Samples which produced a Ct value of <35 on the Cruzi 1/2/3 qPCR and were successfully typed on the SL-IR qPCR were considered positive for *T*. *cruzi* infection. Each PCR included negative controls (water), as well as positive controls of DNA extracted from *T*. *cruzi* Sylvio X10 CL4 (ATCC 50800, American Type Culture Collection [ATCC], Manassas, VA; DTU TcI), *T*. *cruzi*-positive (DTU TcIV) *T*. *sanguisuga* collected from Lee County, Texas, and *T*. *cruzi* Y strain (ATCC 50832, ATCC; DTU TcII).

A subset of five triatomines was processed to determine sources of recent bloodmeals. Hindgut DNA was subjected to PCR using previously published ‘herp’ primers [61,62], direct Sanger sequenced, and compared to existing sequences in GenBank using BLAST as previously described [63]. Negative controls (water) and a positive control of DNA extracted from white-tailed deer (*Odocoileus virginianus*) heart tissue were included in each PCR batch. A bloodmeal identification attempt was considered successful when there was a match with ≥98% BLAST identity in the GenBank database.

## Results

### Dog health monitoring

Blood samples were collected from Ziza immediately before (October 2017) and after (December 2017) the field work. The anti-*T*. *cruzi* IFA titer value prior to field work was 1280 and after field work was 320. A blood sample collected prior to fieldwork had a Ct value of 29 on the Cruzi 1/2/3 PCR, but the DTU was unable to be determined using the SL-IR PCR. After field work, the dog was PCR-negative. A 48-hour Holter monitor placed during the final week of this project documented a sinus arrhythmia with an average heart rate of 82 beats/min and one couplet of ventricular premature contractions, as well as periods of sinus arrest with the maximum lasting 5.3 seconds. Ziza was not symptomatic for the arrhythmia, and clinical recommendations were no treatment and continued monitoring.

### Triatomine collections

The dog/handler team visited a total of 17 field sites across Texas over a period of six weeks during October—December 2017. Triatomines were collected at 8 sites (Fig 1), from a total of 15 searching locations at those 8 sites. Across the 17 sites, an additional 76 locations were searched with no triatomines encountered. An estimated 86 hours were spent actively searching, including 35 hours of dog and handler working together, and 51 hours of humans searching without dog during the day. Search time did not include time spent getting the dog out and ready, putting the dog away, breaks, or gathering and placing bugs into the containers after finding them.

A total of 110 live triatomines were collected (S1 Table), including 107 nymphs (97.2%) and 3 adults (2.7%; two *T*. *protracta* and one *T*. *sanguisuga*). There were 3 instances in which a single triatomine was detected; all other detections were of triatomines in groups: 2 groups of 2 triatomines each, 2 groups of 3 triatomines, 3 groups of 6 triatomines, 1 group of 10 triatomines, 2 groups of 14 triatomines, 1 group of 17 triatomines (Fig 2C), and 1 group of 24 triatomines. A total of 51 (46%) of the triatomines from 5 sites were located by the dog without assistance from other team members; and a total of 9 (8%) triatomines from 2 sites were located by the dog with human assistance. A total of 50 (45%) specimens from 3 sites were located by human team members only (Table 1). The majority of the human-only collections came from a single site (San Antonio 1) from harborage locations previously known to contain triatomines. All other specimens collected by the dog and/or humans were collected from newly discovered harborage locations. We found approximately 0.98 triatomines per hour when only humans were conducting searches, even at the site (San Antonio 1) that was known to harbor triatomines. When working with the dog, we found approximately 1.71 triatomines per hour in a wide variety of microhabitats.

**Table 1:** Search success by detection type and calculation of specimens found per hours searched.

Detection type	Nymphs found	Adults found	Searching hours	Triatomines per hour searched
Dog only	50	1 *T*. *protracta*	35	1.71
Dog with human assist	8	1 *T*. *protracta*		
Humans only	49	1 *T*. *sanguisuga*	51	0.98

Detections were classified as: 1) triatomines located solely by dog; 2) triatomines located by dog with a human assist; 3) triatomines located by human team members. Searching hours were grouped by those during which the dog was active and those during which only humans were searching (generally while the dog was resting).

Searches were performed in habitats with a diversity of potential triatomine locations, including wood piles, stacked bricks, sheet metal on ground, stone, cactus, along walls, inside sheds, underneath potted plants, and in farming equipment. However, wood piles (see example in Fig 2D; specifically natural wood or railroad ties but not treated lumber) comprised the predominant location type in which nymphs were found (99%; 106 of 107 nymphs were found in these habitats). One exception was a single nymph found by a human in a trash pile consisting primarily of metal and plastic. All three adult specimens were found with nymphs (all found in woodpiles). Many specimens were found on the underside of a log or railroad tie, usually at the bottom of a moderate to large sized pile. Nymphs were typically found in shady areas with moist soil and other invertebrates, such as striped bark scorpions (*Centruroides vittatus*), sand cockroaches (*Arenivaga* sp.), ant lions (*Myrmeleon* spp.), and pillbugs (*Armadillidium* sp., *Cubaris* spp.). Specimens were often found in or near locations with dog kennels or evidence of rodents (feces and nesting materials). Distances from collections to nearby houses/dwellings ranged from 13 to 116m away (S1 Table). On more than one occasion, toads (*Bufo* spp.) were found in areas where triatomine nymphs and rodent feces were also found.

Although nymphal stages were not determined for individual samples, it was generally observed that most of the nymphs were 4^th^ or 5^th^ instar nymphs, with occasional 3^rd^ instar nymphs. Of the 107 nymphs collected, 12 were subjected to amplification of the cytochrome b genetic region–nine samples were molecularly identified as *T*. *gerstaeckeri*. Forty-nine nymphs collected from October 30, 2017 through November 29, 2017 at the San Antonio 1 and San Antonio 2 sites were combined in the same colony tub; these collections had ranged from findings of 1–14 triatomines from a total of 7 different locations at these sites. Of these 49 nymphs, all except two eventually molted to adults morphologically identified as *T*. *gerstaeckeri*, while the remaining nymphs molted into one adult morphologically identified as *T*. *indictiva* and one adult morphologically identified as *T*. *sanguisuga*. Fourteen nymphs collected November 17, 2017 from the Edinburgh 1 site were combined in the same colony tub, and all eventually molted to adults identified as *T*. *gerstaeckeri*. Other collections were tracked individually until they molted into adults and then were combined into new containers for future projects. In total, 9 nymphs were identified molecularly as *T*. *gerstaeckeri*, 3 nymphs were attempted but unable to be identified molecularly, 92 molted from nymphs into adult *T*. *gerstaeckeri*, 1 molted from nymph into adult *T*. *indictiva*, 1 molted from nymph into adult *T*. *sanguisuga*, and 1 was not tracked individually to determine species.

Of the 107 nymphs collected, 103 were tested for *T*. *cruzi* infection (S2 Table), and 28 (27.2%) were positive for *T*. *cruzi*; 23 were infected with *T*. *cruzi* DTU TcI, 3 were infected with TcIV, and 2 had mixed TcI/TcIV infections (Table 2). Eleven of the 23 nymphs (47.8%) tested from the Atascosa site were positive–this property includes a privately owned dog breeding and training facility. None of the 21 nymphs tested from the Edinburg 1 and 2 sites (neighboring properties) were positive, and 15 of the 50 nymphs (30.0%) tested from the San Antonio 1 and 2 sites were positive. Of the three adult triatomines collected, 2 were *T*. *protracta* and 1 was *T*. *sanguisuga*. All three adult triatomines were tested (S2 Table), and 2 were positive for *T*. *cruzi* TcI, including one *T*. *sanguisuga* and one *T*. *protracta* (Table 2). A subset of 5 specimens (4 nymphs– 3 positive for *T*. *cruzi* TcI and 1 negative—and the one TcI *T*. *cruzi*-positive adult *T*. *protracta*) were sacrificed and subjected to dissection, bloodmeal scoring, and bloodmeal analysis, which revealed sequences with ≥98% BLAST matches to *Neotoma micropus* (Southern plains woodrat; found in 1 *T*. *protracta* adult, which had also tested positive for *T*. *cruzi* TcI, from the San Antonio 1 site), *Sylvilagus floridanus* (eastern cottontail; found in 1 *T*. *gerstaeckeri* nymph, which had tested negative for *T*. *cruzi*, from the Edinburgh 1 site), and *Didelphis virginiana* (opossum; found in 3 *T*. *gerstaeckeri* nymphs collected from the same Atascosa site, which had all tested positive for *T*. *cruzi* TcI) (Table 3).

**Table 2:** *T*. *cruzi* infection in triatomines collected by a scent detection dog and her human handler.

Site	Triatomine nymphs found	Triatomine nymphs tested	Number nymphs positive (%)
Atascosa	24	23	11 (47.8%)
Brackettville	6	6	0 (0%)
Edinburgh 1	17	15	0 (0%)
Edinburgh 2	6	6	0 (0%)
Kerrville	1	1	0 (0%)
San Antonio 1 and 2^a^	51	50	15 (30.0%)
Uvalde 2^b^	2	2	2 (100%)
Total	107	103	28 (27.2%)

From October to December 2017, a total of 110 triatomines (107 nymphs and 3 adults) were collected at 8 sites across Texas. A subset was tested for *T*. *cruzi* DNA from either fecal spots or hind gut dissections.

^a^ Triatomines from these sites were combined in the colony and not followed individually (S1 and S2 Tables). In addition to the nymphs in this table, two adults (one *T*. *protracta* and one *T*. *sanguisuga*), both of which were positive for *T*. *cruzi* DNA, DTU TcI, were collected from these sites.

^b^ In addition to the nymphs in this table, one adult *T*. *protracta*, which was *T*. *cruzi* negative, was collected from this site.

**Table 3:** Bloodmeal analysis of triatomines found by a scent detection dog.

ID	Blood meal score	Species and life stage	Found by	Site	Location description	*T*. *cruzi* status (DTU)	Bloodmeal source
D+Z 017	3	*T*. *protracta* adult	Dog with human assist	San Antonio 1	Small pile of railroad ties with thorns and trees	Positive (TcI)	*Neotoma micropus* (Southern plains woodrat)
PS 3395A	4	*Triatoma gerstaeckeri* nymph	Dog	Edinburgh 1	Pile of railroad ties in livestock pen with brush piles	Negative	*Sylvilagus floridanus* (eastern cottontail)
D+Z 020A^a^	4	*Triatoma gerstaeckeri* nymph	Dog	Atascosa	Pile of railroad ties in a ditch	Positive (TcI)	*Didelphis virginiana* (opossum)
D+Z 020B^a^	5	*Triatoma gerstaeckeri* nymph	Dog	Atascosa	Pile of railroad ties in a ditch	Positive (TcI)	*Didelphis virginiana* (opossum)
D+Z 020C^a^	5	*Triatoma gerstaeckeri* nymph	Dog	Atascosa	Pile of railroad ties in a ditch	Positive (TcI)	*Didelphis virginiana* (opossum)

A subset of 5 triatomines were subjected to bloodmeal analysis to determine recent bloodmeal hosts. The three nymphs with opossum findings were all found in the same nest area. The species of the nymphs was determined via PCR, Sanger sequencing, and BLAST search comparisons of the cytochrome b gene

^a^These three nymphs were found in the same location.

## Discussion

Triatomine vector control by source (habitat) reduction is challenging because triatomines are nidicolous and cryptic. We trained a scent detection dog to reveal sylvatic locations of triatomines across Texas. The dog and handler team collected a total of 110 triatomines of four species across 8 sites. *T*. *cruzi* infection was found in 27% of nymphs and 2 of 3 adult triatomines tested; this nymphal infection prevalence was higher than what we previously reported in a state-wide community science initiative in which 11.3% of 53 nymphs were infected [64]. Bloodmeals from Southern plains woodrat, eastern cottontail, and Virginia opossum were detected in a subset of 5 specimens. This study expands the proof-of-concept work conducted by a Paraguay team and their triatomine detection dog, Nero, [35] to show that a trained scent detection dog can be an asset for revealing multiple species of triatomines; in our case, increasing the rate of triatomine collection compared to human-only efforts at the sites.

Due to concerns for the risk of *T*. *cruzi* transmission to scent detection dogs, we trained an asymptomatic *T*. *cruzi*-infected dog. Our rapid success in training the dog was partially owed to the dog’s extensive prior training as an explosives detection dog. While Ziza repeatedly tested seropositive and PCR positive for *T*. *cruzi* after her initial diagnosis, she remained asymptomatic after her diagnosis in June of 2016. The dog’s positive serology and contradictory PCR results from pre- and post-field work (positive in October 2017; negative in December 2017) are reflective of the difficulty of detection of low levels of circulating *T*. *cruzi* even when using relatively sensitive PCR protocols [52,54,65]. Fluctuating PCR results in chronically-infected dogs have been documented as a main reason to use serology for a diagnosis [65]. Effects of reinfection on dog serology, PCR results, and disease progression have rarely been studied; one study documented progressively decreasing parasitemia and increasing antibody levels after reinfections [66]. The diagnosis, lack of symptoms, and previous detection experience made this dog an ideal candidate for triatomine detection.

The number of nymphs collected using the scent detection dog was remarkable given our research team’s previous experience attempting to collect triatomines from sylvatic habitats including animal nests. Submissions of nymphs to community science programs in the US are rare (two separate groups found that ~96% of submissions by community scientists were adult triatomines [18,19]), perhaps because of nymphs’ inability to fly to locations where humans are likely to encounter them and also because of their smaller and more cryptic appearance compared to the adult triatomines. Triatomines found in the US are well-documented as occurring in woodrat nests [28,67,68], which are distinctively built and recognizable [28,69]; using a dog helps to eliminate human search image and potential sampling bias and helps identify less easily-observed triatomine harborage sites, including sites where other wildlife species dwell, as revealed by the bloodmeal analysis findings that included eastern cottontail and Virginia opossum blood (Table 3).

Strikingly, and a testament to the dog’s high standard for indicating, in our training and study, the dog did not falsely indicate at any locations (i.e., every time she indicated, the human was able to find a triatomine); however, it is unknown how many times the dog may have not signaled when in fact there was a triatomine in the area. In some cases, the dog lingered at a location without indicating, and when the human returned later to search, a triatomine was found. In this study, we did not count those detections as dog or dog assisted because there was not a clear indication. We found approximately 0.98 triatomines per hour when only humans were conducting searches, driven mostly by collections at a particular site (San Antonio 1) that was known to harbor triatomines. When working with the dog, we found approximately 1.71 triatomines per hour in a wide variety of microhabitats. This is compared to our previous calculations of 3.8 bugs per hour when actively/destructively sampling animal nest habitats *known* to harbor triatomines, without a dog (woodrat nests in Uvalde County, Texas in July of 2014 and August of 2015) [18]. We had not previously done any other kinds of daytime, human-only searching in areas of unknown triatomine occupancy to which this estimate may be compared. It is important to note that the dog was able to help direct us to triatomine harborage locations that we otherwise would not have detected. This enhanced detection would be especially valuable for regions where triatomines are either not known to exist or only known to exist due to the collection of an adult specimen (e.g., [70]).

One potential limitation of the training was that only *T*. *gerstaeckeri* samples were available for training, and it is unknown whether the dog was able to detect other species. For example, there were two situations where adult *T*. *protracta*–one found by the dog only and one found by the dog with human assist–were found with one or two nymphs. In one case, the two nymphs were later identified as *T*. *gerstaeckeri*, so it is unknown whether Ziza would have detected other species alone. *T*. *gerstaeckeri* is the most commonly encountered triatomine throughout much of Texas [18], so this was not a major limitation for this pilot work. It was interesting to find an adult *T*. *protracta* with two *T*. *gerstaeckeri* nymphs. There is a paucity of information regarding how often multiple *Triatoma* species found in the US may inhabit the same animal nests. In addition, there was one nymph that molted into an adult *T*. *indictiva* and one nymph that molted into an adult *T*. *sanguisuga*, but these were discovered after they had been combined with other collections of *T*. *gerstaeckeri* in the colony; it is unknown whether either of these nymphs was found without any *T*. *gerstaeckeri* nymphs. The finding of only nymphs in late fall is consistent with the previously described life history for *T*. *gerstaeckeri*, which described them overwintering as nymphs and developing into adults the following spring [71].

The 27% *T*. *cruzi* infection prevalence in nymphs we found in this work is higher than findings of 11% from our previous work [64]. Additional laboratory work, such as including known *T*. *cruzi* negative fecal spots and triatomines in extraction and PCR as negative controls, as well as use of internal amplification controls, could strengthen the testing results. As more information about vector-host relationships emerges from additional collections of nymphs from sylvatic habitats, it will be interesting to see how nymphal infection prevalence is affected by hosts, including whether certain hosts are reservoir species. Nearly half of the triatomines (51 nymphs and 2 adults) in this study were collected from areas (San Antonio 1 and 2) without permanent structures, but where people regularly sleep outdoors with minimal shelter for the purposes of military training; the two adult triatomines and 15 of the 50 nymphs (30%) tested from these sites were positive for *T*. *cruzi*. Although transmission risk may exist in this setting, an epidemiological study found no human cases of Chagas diseases clearly attributed to soldiers using these areas [72], perhaps because of the inefficiency of stercorarian transmission when a limited number of human-triatomine encounters occur [73]. The military personnel training at these sites are provided bed netting for their cots to sleep overnight, and the vegetation and accumulated debris serving as potential habitat for rodents and small animals has been removed from proximity to the sleeping areas.

Although rare in the US, triatomine colonization and/or reinfestation from sylvatic sources threatens triatomine control in domestic and peridomestic settings throughout the Americas [46,74–78]. In the current study, the nearest houses/dwellings to collections were 13-86m away. In a previous study of over 2,300 triatomines collected by community scientists and members of our research team in the US, less than 2% of samples were nymphs collected from in residences [18]. These data suggest that nymphal dispersion from sylvatic to domestic settings in the US may be rare. Likewise, domestic colonization by triatomines in the US is also rare [14,15,79]. In contrast, we found a concerningly high 48% infection prevalence in nymphs collected from the Atascosa site, a property that is home to a privately owned dog breeding and training facility with *T*. *cruzi* infection in many of its several dozen dogs [80]. These are some of the first collections of nymphs from near dog kennels, as studies have found mainly adults in kennel settings [4,5,7,8,67,81]. Although control of triatomines in sylvatic habitats would be complex, information about reservoirs and infection could guide surveillance and control strategies focused on intercepting triatomines in peridomestic and domestic settings where they pose a transmission risk to people and domestic animals.

Although only a small subset of 5 triatomines were subjected to bloodmeal analysis, the results revealed a new host species and confirmed previously reported host species. We believe this is the first report of the eastern cottontail as a bloodmeal source. Another species of cottontail—*Sylvilagus audubonii*—blood has been detected in triatomines, including triatomines infected with *T*. *cruzi* [82]. The discovery of this triatomine blood source was due to the dog’s ability to detect nymphs in animal nests. The ability to detect bloodmeal sources that sustain nymphs until they can disperse as adults is a strength of this study; this information can help guide our understanding of any species that are particularly important to the early triatomine life cycle and allow for targeted interruption of the life cycle. Woodrats have previously been reported as bloodmeal sources in triatomines collected in Texas [67], and the findings of triatomines in woodrat nests are well-documented [69], as well as findings of *T*. *cruzi* in this bloodmeal host [83]. In our study, the triatomine with evidence of woodrat blood was infected with *T*. *cruzi* DTU TcI; previous work has revealed woodrats infected with DTUs TcI and TcIV [83,84]. Opossum blood has been previously detected in triatomines [85], and opossums are well-known reservoirs of *T*. *cruzi* [3,79], specifically DTU TcI [86–91]. As expected from this DTU-host association, the nymphs in this study with evidence of opossum blood were infected with *T*. *cruzi* DTU TcI. A limitation of this study was the use of PCR and Sanger sequencing, which does not allow for detection of multiple bloodmeal sources, compared to other methods that may detect mixed bloodmeal sources [85,92–94].

Methods to track triatomines, their hosts, and *T*. *cruzi* reservoirs species in sylvatic locations range from intensive efforts of manual searching [8,28,95] to innovative methods such as telemetry of insects [96,97] and spool-and-line techniques to follow wildlife hosts [98–102]. While manual searching can be aided by recognition of conspicuous nesting sites, telemetry and spool-and-line methods rely on capturing, tagging, releasing, and following the triatomines or their hosts. Although using a scent detection dog requires access to a specialized dog and an investment in training, such dogs may afford more opportunities to detect triatomines in less conspicuous nesting locations without the need to capture and track individual animals. Of note, many of the triatomines in the current study were collected from under piles of wood or railroad ties. These piles likely serve as locations where small prey animals can nest safely away from predators. The semi-protected areas under the wood also likely allowed for triatomine scent to pool and be more detectable to the dog. It may be that groups of nymphs are more easily detected than single adults moving through an area because of the scent pool that forms around the cluster of nymphs.

Multiple hazards were encountered during this field work that should be considered when planning to conduct dog scent detection work. Most relevant hazards were the sun and heat, venomous snakes, venomous arthropods, and prickly pear cactus (*Opuntia* spp., the spines of which may pose a physical hazard). Piles of scrap and sheet metal, old lumber with exposed nails, broken glass, barbed wire, and exposed metal fencing were also commonly encountered. To mitigate potential hazards, the dog was always worked on lead. A two-person team was more effective than the dog handler working alone with the dog, as handler collection of triatomines was difficult when the dog was actively excited about a detection. For personal protection, the handler wore long pants, a long-sleeved shirt, thick-soled boots, snake guards, a hat, and gloves; the dog wore a snake proof vest and a brightly colored vest, as rattlesnakes were a danger and work was conducted in areas with active hunting. Handlers should speak with their veterinarian about whether a rattlesnake vaccine is appropriate pre-exposure prophylaxis. Working during the heat of the day when temperatures exceed 27°C (80°F) in the direct sun was avoided whenever possible. A variety of potential distractions–including animals, animal feces, gunshot sounds, and swimming pools–can be anticipated during field work and should be addressed during dog training. During fieldwork, we realized that a ‘sit at source’ indication was not the best indication for a dog doing this type of work. In many cases, the dog was unable to sit when making a detection because most detections were on the top or side of large wood piles where debris posed a sliding or otherwise unstable hazard. A bark or focused indication may be a better choice. In contrast to ‘sit at source,’ digging was useful as a secondary indication.

In addition to careful consideration of local safety issues, others interested in training a dog for triatomine detection will find it most fruitful to work with an experienced scent detection dog and skilled trainer. Ziza’s background and training as a detection dog made her an ideal candidate, as 19 hours of training were sufficient for her to recognize and signal detections of triatomines. In a 2008 report, the amount spent by various government agencies to procure an untrained dog ranged from $3,500–4,500 [103]; and in 2021 the costs associated with training a TSA scent detection dog and handler ranged from $33,000–46,000 [104]. Scent detection dogs can be cost-prohibitive to include in many kinds of studies [105]. Both the method of training and the nature of the task may result in nuances in how the dog signals. Continuous training of the dog/human pair, particularly blinded training and a handler’s attentiveness to nuances in the dog’s signaling is key to the success of this method.

Although triatomines occur in 30 states in the US [106], many states have relatively few documented occurrences of triatomines. Local public health agencies and triatomine researchers frequently attempt to collect triatomines from areas where human or animal cases of Chagas have occurred or where collections have been made in the past. Often considerable search effort can yield low success rates [107], and it is likely that many search efforts for triatomines that did not yield collections remain unpublished. Having a scent detection dog trained specifically on triatomines would be a valuable tool in many contexts in the US and beyond. Detection of nidicolous triatomine nymphs in sylvatic locations was successful using a scent detection dog, and future work can help to elucidate key areas for vector control for disease prevention.

## Supporting information

S1 Table Field sites across Texas where triatomines were encountered by a scent detection dog and/or humans. The triatomine habitat is described along with notes about the proximity of insects to human dwellings. The triatomine species, *Trypanosoma cruzi* infection status, and bloodmeal hosts of the triatomines are indicated.
(XLSX)

S2 Table Results of *Trypanosoma cruzi* testing of triatomines across Texas field sites in relation to insect life stage. The discrete taxonomic unit (DTU) of *T*. *cruzi* is indicated.
(XLSX)

## References

[1] World Health Organization (2015) Chagas disease in Latin America: an epidemiological update based on 2010 estimates. Wkly Epidemiol Rec 6: 33–44. 25671846

[2] Bern C, Montgomery SP (2009) An estimate of the burden of Chagas disease in the United States. Clin Infect Dis 49: e52–4. doi: 10.1086/605091 19640226

[3] Hodo CL, Hamer SA (2017) Toward an ecological framework for assessing reservoirs of vector-borne pathogens: wildlife reservoirs of *Trypanosoma cruzi* across the southern United States. ILAR J 58: 379–392. doi: 10.1093/ilar/ilx020 29106561 PMC6019048

[4] Williams G, Adams LG, Yaeger RG, McGrath RK, Read WK, et al. (1977) Naturally occurring trypanosomiasis (Chagas’ disease) in dogs. J Am Vet Med Assoc 171: 171–177. 407202

[5] Curtis-Robles R, Snowden KF, Dominguez B, Dinges L, Rodgers S, et al. (2017) Epidemiology and molecular typing of *Trypanosoma cruzi* in naturally-infected hound dogs and associated triatomine vectors in Texas, USA. PLoS Negl Trop Dis 11: e0005298. doi: 10.1371/journal.pntd.0005298 28095511 PMC5287457

[6] Tenney TD, Curtis-Robles R, Snowden KF, Hamer SA (2014) Shelter dogs as sentinels for *Trypanosoma cruzi* transmission across Texas. Emerg Infect Dis 20: 1323–1326. doi: 10.3201/eid2008.131843 25062281 PMC4111162

[7] Meyers AC, Meinders M, Hamer SA (2017) Widespread *Trypanosoma cruzi* infection in government working dogs along the Texas-Mexico border: Discordant serology, parasite genotyping and associated vectors. PLoS Negl Trop Dis 11: 1–19. doi: 10.1371/journal.pntd.0005819 28787451 PMC5560752

[8] McPhatter L, Lockwood N, Roachell W, Mahmood F, Hoffman L, et al. (2012) Vector surveillance to determine species composition and occurrence of *Trypanosoma cruzi* infection at three military installations in San Antonio, Texas. Army Med Dep J 07: 12–21. 22815160

[9] Rowland ME, Maloney J, Cohen S, Yabsley MJ, Huang J, et al. (2010) Factors ssociated with *Trypanosoma cruzi* exposure among domestic canines in Tennessee. J Parasitol 96: 547–551. doi: 10.1645/GE-2299.1 20557201

[10] Bradley K, Bergman D, Woods J, Crutcher J, Kirchhoff L (2000) Prevalence of American trypanosomiasis (Chagas disease) among dogs in Oklahoma. J Am Vet Med Assoc 217: 1853–1857. doi: 10.2460/javma.2000.217.1853 11132891

[11] Bern C, Kjos S, Yabsley MJ, Montgomery SP (2011) *Trypanosoma cruzi* and Chagas’ Disease in the United States. Clin Microbiol Rev 24: 655–681. doi: 10.1128/CMR.00005-11 21976603 PMC3194829

[12] Schofield CJ, Dias JCP (1999) The Southern Cone Initiative against Chagas Disease. Adv Parasitol 42: 1–27. doi: 10.1016/s0065-308x(08)60147-5 10050271

[13] Dias J, Silveira A, Schofield C (2002) The impact of Chagas disease control in Latin America—A review. Mem Inst Oswaldo Cruz Rio Janeiro 97: 603–612. doi: 10.1590/s0074-02762002000500002 12219120

[14] Lent H, Wygodzinsky PW (1979) Revision of the Triatominae (Hemiptera, Reduviidae), and their significance as vectors of Chagas’ Disease. Bull Am Museum Nat Hist 163: 123–520.

[15] Klotz SA, Shirazi FM, Boesen K, Beatty NL, Dorn PL, et al. (2016) Kissing bug (*Triatoma* spp.) intrusion into homes: Troublesome bites and domiciliation. Environ Health Insights 10: 45–49. doi: 10.4137/EHI.S32834 27042091 PMC4807888

[16] Kjos SA, Snowden KF, Olson JK (2009) Biogeography and *Trypanosoma cruzi* infection prevalence of Chagas disease vectors in Texas, USA. Vector-Borne Zoonotic Dis 9: 41–50. doi: 10.1089/vbz.2008.0026 18800865

[17] Wozniak EJ, Lawrence G, Gorchakov R, Alamgir H, Dotson E, et al. (2015) The biology of the triatomine bugs native to south central Texas and assessment of the risk they pose for autochthonous Chagas disease exposure. J Parasitol 101: 520–528. doi: 10.1645/15-748 26168214

[18] Curtis-Robles R, Hamer SA, Lane S, Levy MZ, Hamer GL (2018) Bionomics and spatial distribution of triatomine vectors of *Trypanosoma cruzi* in Texas and other southern states, USA. Am J Trop Med Hyg 98: 113–121. doi: 10.4269/ajtmh.17-0526 29141765 PMC5928729

[19] Reisenman CE, Savary W, Cowles J, Gregory TL, Hildebrand JG (2012) The distribution and abundance of triatomine insects, potential vectors of Chagas Disease, in a metropolitan area in southern Arizona, United States. J Med Entomol 49: 1254–1261. doi: 10.1603/me12139 23270152

[20] Ryckman RE (1986) The vertebrate hosts of the Triatominae of North and Central America and the West Indies (Hemiptera: Reduviidae: Triatominae). Bull Soc Vector Ecol 11: 221–241.

[21] Kofoid CA, McCulloch I (1916) On *Trypanosoma triatomae*, a new flagellate from a hemipteran bug from the nests of the wood rat *Neotoma fuscipes*. Univ Calif Publ Zool 16: 113–126.

[22] Mehringer PJ, Wood SF (1958) A resampling of wood rat houses and human habitations in Griffith Park, Los Angeles, for *Triatoma protracta* and *Trypanosoma cruzi*. Bull South Calif Acad Sci 57: 39–46.

[23] Wood SF, Wood FD (1961) Observations on vectors of Chagas’ disease in the United States III. New Mexico. Am J Trop Med Hyg 10: 155–165. 13786516 10.4269/ajtmh.1961.10.155

[24] Ryckman RE, Ryckman AE (1965) Epizootiology of *Trypanosoma cruzi* in Southwestern North America: Part I: New collection records and hosts for *Trypanosoma cruzi* Chagas (Kinetoplastida: Trypanosomidae) (Hemiptera: Triatominae). J Med Entomol 2: 87–89. 14302117 10.1093/jmedent/2.1.87

[25] Wood FD (1934) Natural and experimental infection of *Triatoma protracta* Uhler and mammals in California with American human trypanosomiasis. Am J Trop Med Hyg 14: 497–517.

[26] Wood SF (1937) A new locality for *Trypanosoma cruzi* Chagas in California. Science 87: 366–367. doi: 10.1126/science.87.2260.366 17808585

[27] Pippin W (1970) The biology and vector capability of *Triatoma sanguisuga texana* Usinger and *Triatoma gerstaeckeri* (Stål) compared with *Rhodnius prolixus* (Stål) (Hemiptera: Triatominae). J Med Entomol 7: 30–45. 4907991 10.1093/jmedent/7.1.30

[28] Ekkens D (1984) Nocturnal flights of *Triatoma* (Hemiptera: Reduviidae) in Sabino Canyon, Arizona II. *Neotoma* lodge studies. J Med Entomol 21: 140–144.

[29] Browne C, Stafford K, Fordham R (2006) The use of scent-detection dogs. Ir Vet J 59: 97–104.

[30] Kantele A, Paajanen J, Turunen S, Pakkanen SH, Patjas A, et al. (2022) Scent dogs in detection of COVID-19: triple-blinded randomised trial and operational real-life screening in airport setting. BMJ Glob Heal 7: e008024. doi: 10.1136/bmjgh-2021-008024 35577391 PMC9108438

[31] Otto CM, Sell TK, Veenema T, Hosangadi D, Vahey RA, et al. (2021) The promise of disease detection dogs in pandemic response: Lessons learned from COVID-19. Disaster Med Public Health Prep: 1–6. doi: 10.1017/dmp.2021.183 34099088 PMC8460421

[32] Guest C, Pinder M, Doggett M, Squires C, Affara M, et al. (2019) Trained dogs identify people with malaria parasites by their odour. Lancet Infect Dis 19: 578–580. doi: 10.1016/S1473-3099(19)30220-8 31122774

[33] Lindsay SW, Pinder M, Squires C, Doggett M, Kasstan B, et al. (2018) Can medical-detection dogs identify people with malaria parasites? ASTMH 67th Annual Meeting Abstracts. Vol. 99. Available from: https://www.astmh.org/ASTMH/media/Documents/2018-Abstract-Book-FINAL-11-13.pdf.

[34] Moser E, McCulloch M (2010) Canine scent detection of human cancers: A review of methods and accuracy. J Vet Behav Clin Appl Res 5: 145–152. doi: 10.1016/j.jveb.2010.01.002

[35] Gonder-Frederick LA, Grabman JH, Shepard JA (2017) Diabetes Alert Dogs (DADs): An assessment of accuracy and implications. Diabetes Res Clin Pract 134: 121–130. doi: 10.1016/j.diabres.2017.09.009 28974470 PMC5723560

[36] Pfiester M, Koehler PG, Pereira RM (2008) Ability of bed bug-detecting canines to locate live bed bugs and viable bed bug eggs. J Econ Entomol 101: 1389–1396. doi: 10.1603/0022-0493(2008)101[1389:aobbct]2.0.co;2 18767752

[37] Cooper R, Wang C, Singh N (2014) Accuracy of trained canines for detecting bed bugs (Hemiptera: Cimicidae). J Econ Entomol 107: 2171–2181. doi: 10.1603/EC14195 26470083

[38] Brooks SE, Oi FM, Koehler PG (2003) Ability of canine termite detectors to locate live termites and discriminate them from non-termite material. J Econ Entomol 96: 1259–1266. doi: 10.1093/jee/96.4.1259 14503599

[39] Wallner WE, Ellis TL (1976) Olfactory detection of gypsy moth pheromone and egg masses by domestic canines. Environ Entomol 5: 183–186. doi: 10.1093/ee/5.1.183

[40] Lee DH, Cullum JP, Anderson JL, Daugherty JL, Beckett LM, et al. (2014) Characterization of overwintering sites of the invasive brown marmorated stink bug in natural landscapes using human surveyors and detector canines. PLoS One 9: e91575. doi: 10.1371/journal.pone.0091575 24717734 PMC3981664

[41] Essler JL, Kane SA, Collins A, Ryder K, DeAngelo A, et al. (2021) Egg masses as training aids for spotted lanternfly *Lycorma delicatula* detection dogs. PLoS One 16: e0250945. doi: 10.1371/journal.pone.0250945 33939739 PMC8092771

[42] Welch JB (1990) A detector dog for screwworms. J Econ Entomol 83: 1932–1934. 2258514 10.1093/jee/83.5.1932

[43] Samer A, Permunian R, Gakuya F, Mutinda M, Soriguer RC, et al. (2012) Sarcoptic-mange detector dogs used to identify infected animals during outbreaks in wildlife. BMC Vet Res 8: 110. doi: 10.1186/1746-6148-8-110 22776804 PMC3407749

[44] Rolón M, Vega MC, Román F, Gómez A, de Arias AR, et al. (2011) First report of colonies of sylvatic *Triatoma infestans* (Hemiptera: Reduviidae) in the Paraguayan Chaco, using a trained dog. PLoS Negl Trop Dis 5: e1026. doi: 10.1371/journal.pntd.0001026 21572522 PMC3086807

[45] Provecho YM, Sol Gaspe M, Del Pilar Fernández M, Gürtler RE, Byrd J (2017) House reinfestation with *Triatoma infestans* (Hemiptera: Reduviidae) after community-wide spraying with insecticides in the Argentine Chaco: A multifactorial process. J Med Entomol 54: 646–657. doi: 10.1093/jme/tjw224 28399199

[46] de Arias AR, Messenger LA, Rolon M, Vega MC, Acosta N, et al. (2022) Dynamics of *Triatoma infestans* populations in the Paraguayan Chaco: Population genetic analysis of household reinfestation following vector control. PLoS One 17: 1–23. doi: 10.1371/journal.pone.0263465 35143523 PMC8830694

[47] de Arias AR, Monroy C, Guhl F, Sosa-Estani S, Santos WS, et al. (2021) Chagas disease control-surveillance in the Americas: The multinational initiatives and the practical impossibility of interrupting vector-borne *Trypanosoma cruzi* transmission. Mem Inst Oswaldo Cruz 116: 1–15. doi: 10.1590/0074-02760210130 35830010 PMC9261920

[48] Curtis-Robles R, Wozniak EJ, Auckland LD, Hamer GL, Hamer SA (2015) Combining public health education and disease ecology research: using citizen science to assess Chagas disease entomological risk in Texas. PLoS Negl Trop Dis 9: e0004235. doi: 10.1371/journal.pntd.0004235 26658425 PMC4687635

[49] QGIS Association (2023) QGIS Geographic Information System. Available from: http://www.qgis.org/.

[50] State of Texas (n.d.) Texas Open Data Portal [Counties]. Available from: https://data.texas.gov/dataset/Texas-Counties-Map/48ag-x9aa. Accessed 15 February 2023.

[51] Curtis-Robles R, Zecca IB, Roman-Cruz V, Carbajal ES, Auckland LD, et al. (2017) *Trypanosoma cruzi* (agent of Chagas Disease) in sympatric human and dog populations in “colonias” of the Lower Rio Grande Valley of Texas. Am J Trop Med Hyg 96: 805–814. doi: 10.4269/ajtmh.16-0789 28167589 PMC5392625

[52] Duffy T, Cura CI, Ramirez JC, Abate T, Cayo NM, et al. (2013) Analytical performance of a multiplex Real-Time PCR assay using TaqMan probes for quantification of *Trypanosoma cruzi* satellite DNA in blood samples. PLoS Negl Trop Dis 7: e2000. doi: 10.1371/journal.pntd.0002000 23350002 PMC3547845

[53] Piron M, Fisa R, Casamitjana N, López-Chejade P, Puig L, et al. (2007) Development of a real-time PCR assay for *Trypanosoma cruzi* detection in blood samples. Acta Trop 103: 195–200. doi: 10.1016/j.actatropica.2007.05.019 17662227

[54] Cura CI, Duffy T, Lucero RH, Bisio M, Péneau J, et al. (2015) Multiplex real-time PCR assay using TaqMan probes for the identification of *Trypanosoma cruzi* DTUs in biological and clinical samples. PLoS Negl Trop Dis 9: e0003765. doi: 10.1371/journal.pntd.0003765 25993316 PMC4437652

[55] Skinner BF (1958) Reinforcement today. Am Psychol 13: 94–99. doi: 10.1037/h0049039

[56] Pryor K (1999) Don’t shoot the dog!: The new art of teaching and training. New York: Bantam Books.

[57] Pfeiler E, Bitler B, Ramsey J, Palacios-Cardiel C, Markow T (2006) Genetic variation, population structure, and phylogenetic relationships of *Triatoma rubida* and *T*. *recurva* (Hemiptera: Reduviidae: Triatominae) from the Sonoran Desert, insect vectors of the Chagas’ disease parasite *Trypanosoma cruzi*. Mol Phylogenet Evol 41: 209–221. doi: 10.1016/j.ympev.2006.07.001 16934496

[58] Tamura K, Stecher G, Kumar S (2021) MEGA11: Molecular Evolutionary Genetics Analysis version 11. Mol Biol Evol 38: 3022–3027. doi: 10.1093/molbev/msab120 33892491 PMC8233496

[59] Clark K, Karsch-Mizrachi I, Lipman DJ, Ostell J, Sayers EW (2016) GenBank. Nucleic Acids Res 44: D67–D72. doi: 10.1093/nar/gkv1276 26590407 PMC4702903

[60] Altschul SF, Gish W, Miller W, Myers EW, Lipman DJ (1990) Basic local alignment search tool. J Mol Biol 215: 403–410. doi: 10.1016/S0022-2836(05)80360-2 2231712

[61] Cupp EW, Zhang D, Yue X, Cupp MS, Guyer C, et al. (2004) Identification of reptilian and amphibian blood meals from mosquitoes in an eastern equine encephalomyelitis virus focus in central Alabama. Am J Trop Med Hyg 71: 272–276. doi: 10.4269/ajtmh.2004.71.272 15381805 PMC1351276

[62] Hamer GL, Kitron UD, Goldberg TL, Brawn JD, Loss SR, et al. (2009) Host selection by *Culex pipiens* mosquitoes and West Nile virus amplification. Am J Trop Med Hyg 80: 268–278. doi: 10.4269/ajtmh.2009.80.268 19190226

[63] Curtis-Robles R, Meyers A, Auckland L, Zecca I, Skiles R, et al. (2018) Parasitic interactions among *Trypanosoma cruzi*, triatomine vectors, domestic animals, and wildlife in Big Bend National Park along the Texas-Mexico border. Acta Trop 188: 225–233. doi: 10.1016/j.actatropica.2018.09.002 30205082

[64] Curtis-Robles R, Aukland LD, Snowden KF, Hamer GL, Hamer SA (2018) Analysis of over 1500 triatomine vectors from across the US, predominantly Texas, for *Trypanosoma cruzi* infection and discrete typing units. Infect Genet Evol 58: 171–180. doi: 10.1016/j.meegid.2017.12.016 29269323

[65] Araújo F, Bahia M, Magalhães N, Martins-Filho O, Veloso V, et al. (2002) Follow-up of experimental chronic Chagas’ disease in dogs: Use of polymerase chain reaction (PCR) compared with parasitological and serological methods. Acta Trop 81: 21–31. doi: 10.1016/s0001-706x(01)00196-6 11755429

[66] Machado EMM, Fernandes AJ, Murta SMF, Vitor RWA, Camilo D.J. J, et al. (2001) A study of experimental reinfection by *Trypanosoma cruzi* in dogs. Am J Trop Med Hyg 65: 958–965. 11792006 10.4269/ajtmh.2001.65.958

[67] Kjos SA, Marcet PL, Yabsley MJ, Kitron U, Snowden KF, et al. (2013) Identification of bloodmeal sources and *Trypanosoma cruzi* infection in triatomine bugs (Hemiptera: Reduviidae) from residential settings in Texas, the United States. J Med Entomol 50: 1126–1139. doi: 10.1603/ME12242 24180119 PMC3932564

[68] Shender LA, Lewis MD, Rejmanek D, Mazet JAK (2016) Molecular diversity of *Trypanosoma cruzi* detected in the vector *Triatoma protracta* from California, USA. PLoS Negl Trop Dis 10: e0004291. doi: 10.1371/journal.pntd.0004291 26797311 PMC4721664

[69] Eads RB, Trevino HA, Campos EG (1963) *Triatoma* (Hemiptera: Reduviidae) infected with *Trypanosoma cruzi* in South Texas wood rat dens. Southwest Nat 8: 38–42.

[70] Eggers P, Offutt-Powell TN, Lopez K, Montgomery SP, Lawrence GG (2019) Identification of a *Triatoma sanguisuga* “Kissing Bug”—Delaware, 2018. Morb Mortal Wkly Rep 68: 359. doi: 10.1111/hisn.13197 PMC6476055 30998670

[71] Usinger RL (1944) The Triatominae of North and Central America and the West Indies and their public health significance. US Public Heal Serv Public Heal Bull: 81.

[72] Webber BJ, Pawlak MT, Valtier S, Daniels CC, Tully CC, et al. (2017) Prevalence and seroprevalence of *Trypanosoma cruzi* infection in a military population in Texas. Am J Trop Med Hyg 97: 1477–1481. doi: 10.4269/ajtmh.17-0109 28820695 PMC5817750

[73] Nouvellet P, Dumonteil E, Gourbière S (2013) The improbable transmission of *Trypanosoma cruzi* to human: the missing link in the dynamics and control of Chagas disease. PLoS Negl Trop Dis 7: e2505. doi: 10.1371/journal.pntd.0002505 24244766 PMC3820721

[74] Valente V, Valente S, Noireau F, Carrasco H, Miles M (1998) Chagas disease in the Amazon Basin: association of *Panstrongylus geniculatus* (Hemiptera: Reduviidae) with domestic pigs. J Med Entomol 35: 99–103. doi: 10.1093/jmedent/35.2.99 9538568

[75] Schachter-Broide J, Dujardin J-P, Kitron U, Gürtler RE (2004) Spatial structuring of *Triatoma infestans* (Hemiptera, Reduviidae) populations from northwestern Argentina using wing geometric morphometry. J Med Entomol 41: 643–649. 15311455 10.1603/0022-2585-41.4.643 PMC1351235

[76] Vazquez-Prokopec GM, Cecere MC, Canale DM, Gürtler RE, Kitron U (2005) Spatiotemporal patterns of reinfestation by *Triatoma guasayana* (Hemiptera: Reduviidae) in a rural community of northwestern Argentina. J Med Entomol 42: 571–581. doi: 10.1093/jmedent/42.4.571 16119545 PMC1382187

[77] Ceballos LA, Piccinali R V., Marcet PL, Vazquez-Prokopec GM, Cardinal MV, et al. (2011) Hidden sylvatic foci of the main vector of Chagas disease *Triatoma infestans*: Threats to the vector elimination campaign? PLoS Negl Trop Dis 5: e1365. doi: 10.1371/journal.pntd.0001365 22039559 PMC3201917

[78] Brémond P, Salas R, Waleckx E, Buitrago R, Aliaga C, et al. (2014) Variations in time and space of an Andean wild population of *T*. *infestans* at a microgeographic scale. Parasites and Vectors 7: 1–11. doi: 10.1186/1756-3305-7-164 24708673 PMC3992151

[79] Zeledón R, Beard CB, Dias JP, Leiby DA, Dorn PL, et al. (2012) An Appraisal of the Status of Chagas Disease in the United States. Waltham, USA: Elsevier Inc.

[80] Busselman RE, Meyers AC, Zecca IB, Auckland LD, Castro AH, et al. (2021) High incidence of *Trypanosoma cruzi* infections in dogs directly detected through longitudinal tracking at 10 multi-dog kennels, Texas, USA. PLoS Negl Trop Dis 15: e0009935. doi: 10.1371/journal.pntd.0009935 34758049 PMC8631682

[81] Zeledón R, Montenegro VM, Zeledón O (2001) Evidence of colonization of man-made ecotopes by *Triatoma dimidiata* (Latreille, 1811) in Costa Rica. Mem Inst Oswaldo Cruz 96: 659–660. 11500765 10.1590/s0074-02762001000500012

[82] Gorchakov R, Trosclair LP, Wozniak EJ, Feria PT, Garcia MN, et al. (2016) *Trypanosoma cruzi* infection prevalence and bloodmeal analysis in triatomine vectors of Chagas disease from rural peridomestic locations in Texas, 2013–2014. J Med Entomol 53: 911–918. doi: 10.1093/jme/tjw040 27106934

[83] Charles RA, Kjos S, Ellis AE, Barnes JC, Yabsley MJ (2013) Southern plains woodrats (*Neotoma micropus*) from southern Texas are important reservoirs of two genotypes of *Trypanosoma cruzi* and host of a putative novel *Trypanosoma* species. Vector-Borne Zoonotic Dis 13: 22–30. doi: 10.1089/vbz.2011.0817 23127189 PMC3540927

[84] Herrera CP, Licon MH, Nation CS, Jameson SB, Wesson DM (2015) Genotype diversity of *Trypanosoma cruzi* in small rodents and *Triatoma sanguisuga* from a rural area in New Orleans, Louisiana. Parasit Vectors 8: 1–9. doi: 10.1186/s13071-015-0730-8 25890064 PMC4344744

[85] Klotz SA, Schmidt JO, Dorn PL, Ivanyi C, Sullivan KR, et al. (2014) Free-roaming kissing bugs, vectors of Chagas disease, feed often on humans in the Southwest. Am J Med 127: 421–426. doi: 10.1016/j.amjmed.2013.12.017 24398362 PMC4096837

[86] Clark CG, Pung OJ (1994) Host specificity of ribosomal DNA variation in sylvatic *Trypanosoma cruzi* from North America. Mol Biochem Parasitol 66: 175–179. 7984184 10.1016/0166-6851(94)90052-3

[87] Hodo CL, Wilkerson GK, Birkner EC, Gray SB, Hamer SA (2018) *Trypanosoma cruzi* transmission among captive nonhuman primates, wildlife, and vectors. Ecohealth 15: 426–436. doi: 10.1007/s10393-018-1318-5 29497880 PMC6132415

[88] Roellig DM, Brown EL, Barnabé C, Tibayrenc M, Steurer FJ, et al. (2008) Molecular typing of *Trypanosoma cruzi* isolates, United States. Emerg Infect Dis 14: 1123–1125. doi: 10.3201/eid1407.080175 18598637 PMC2600345

[89] Roellig DM, Savage MY, Fujita AW, Barnabé C, Tibayrenc M, et al. (2013) Genetic variation and exchange in *Trypanosoma cruzi* isolates from the United States. PLoS One 8: e56198. doi: 10.1371/journal.pone.0056198 23457528 PMC3572986

[90] Cura CI, Mejía-Jaramillo AM, Duffy T, Burgos JM, Rodriguero M, et al. (2010) *Trypanosoma cruzi* I genotypes in different geographical regions and transmission cycles based on a microsatellite motif of the intergenic spacer of spliced-leader genes. Int J Parasitol 40: 1599–1607. doi: 10.1016/j.ijpara.2010.06.006 20670628 PMC3081674

[91] Barnabé C, Brisse S, Tibayrenc M (2000) Population structure and genetic typing of *Trypanosoma cruzi*, the agent of Chagas disease: a multilocus enzyme electrophoresis approach. Parasitology 120: 513–526. 10840981 10.1017/s0031182099005661

[92] Gürtler RE, Cecere MC, Vazquez DP, Chuit R, Cohen JE (1996) Host-feeding patterns of domiciliary *Triatoma infestans* (Hemiptera: Reduviidae) in northwest Argentina: seasonal and instar variation. J Med Entomol 33: 15–26. 8906900 10.1093/jmedent/33.1.15

[93] Pizarro JC, Stevens L (2008) A new method for forensic DNA analysis of the blood meal in Chagas disease vectors demonstrated using *Triatoma infestans* from Chuquisaca, Bolivia. PLoS One 3: e3585. doi: 10.1371/journal.pone.0003585 18974787 PMC2570791

[94] Dumonteil E, Ramirez-Sierra MJ, Pérez-Carrillo S, Teh-Poot C, Herrera C, et al. (2018) Detailed ecological associations of triatomines revealed by metabarcoding and next-generation sequencing: Implications for triatomine behavior and *Trypanosoma cruzi* transmission cycles. Sci Rep 8: 4140. doi: 10.1038/s41598-018-22455-x 29515202 PMC5841364

[95] Ocaña-Mayorga S, Lobos SE, Crespo-Pérez V, Villacís AG, Pinto CM, et al. (2018) Influence of ecological factors on the presence of a triatomine species associated with the arboreal habitat of a host of *Trypanosoma cruzi*. Parasites and Vectors 11: 567. doi: 10.1186/s13071-018-3138-4 30373640 PMC6206927

[96] Herrera HM, Lisboa C V., Pinho AP, Olifiers N, Bianchi RC, et al. (2008) The coati (*Nasua nasua*, Carnivora, Procyonidae) as a reservoir host for the main lineages of *Trypanosoma cruzi* in the Pantanal region, Brazil. Trans R Soc Trop Med Hyg 102: 1133–1139. doi: 10.1016/j.trstmh.2008.04.041 18541281

[97] Hamer G, Bejcek J, Valdez E, Curtis-Robles R, Hamer S (2018) A pilot radio telemetry field study of triatomine vectors (Hemiptera: Reduviidae) of the Chagas disease parasite. J Med Entomol 55: 1380–1385. doi: 10.1093/jme/tjy094 29986045 PMC6201830

[98] Miles MA, de Souza AA, Póvoa MM (1981) Mammal tracking and nest location in Brazilian forest with an improved spool-and-line device. J Zool 195: 331–347. doi: 10.1111/j.1469-7998.1981.tb03469.x

[99] Miles MA (1976) A simple locating method of tracking mammals and locating triatomine vectors of *Trypanosoma cruzi* in Amazonian forest. Am J Trop Med Hyg 25: 671–674. 786052 10.4269/ajtmh.1976.25.671

[100] Miles MA, de Souza AA, Póvoa MM (1982) O ecótopo de *Panstrongylus megistus* (Hemiptera, Reduviidae) na floresta do Horto (Rio de Janeiro). Rev Bras Biol 42: 31–35. 6753051

[101] dos Santos JE, Viola MG, Lorosa ES, Machado EM de M, Ruas Neto AL, et al. (2013) Evaluation of natural foci of *Panstrongylus megistus* in a forest fragment in Porto Alegre, State of Rio Grande do Sul, Brazil. Rev Soc Bras Med Trop 46: 575–583. doi: 10.1590/0037-8682-0149-2013 24270248

[102] Alvarado-Otegui JA, Ceballos LA, Orozco MM, Enriquez GF, Cardinal M V., et al. (2012) The sylvatic transmission cycle of *Trypanosoma cruzi* in a rural area in the humid Chaco of Argentina. Acta Trop 124: 79–86. doi: 10.1016/j.actatropica.2012.06.010 22771688 PMC3444808

[103] Office of Inspector General (2008) A Review of U. S. Customs and Border Protection’s Procurement of Untrained Canines OIG-08-46. 31 p.

[104] Transportation Security Administration (2021) TSA Canine Training Center. Available from: https://www.tsa.gov/news/press/factsheets/tsa-canine-training-center. Accessed 13 December 2022.

[105] Orkin JD, Yang Y, Yang C, Yu DW, Jiang X (2016) Cost-effective scat-detection dogs: Unleashing a powerful new tool for international mammalian conservation biology. Sci Rep 6: 1–10. doi: 10.1038/srep34758 27721442 PMC5056371

[106] Bern C, Messenger LA, Whitman JD, Maguire JH (2019) Chagas disease in the United states: A public health approach. Clin Microbiol Rev 33: 1–42. doi: 10.1128/CMR.00023-19 31776135 PMC6927308

[107] Dye-Braumuller KC, Gorchakov R, Gunter SM, Nielsen DH, Roachell WD, et al. (2019) Identification of triatomines and their habitats in a highly developed urban environment. Vector-Borne Zoonotic Dis 19: 265–273. doi: 10.1089/vbz.2018.2352 30571182 PMC6459272

